# Incidental Discovery of Giant Ovarian Cysts During Autopsy. Case Analysis and Literature Review

**DOI:** 10.15388/Amed.2024.31.2.16

**Published:** 2024-12-04

**Authors:** Justė Kazlauskaitė, Sigitas Chmieliauskas, Diana Vasiljevaitė, Sigitas Laima

**Affiliations:** 1 Faculty of Medicine, Vilnius University, Vilnius, Lithuania; 2 Department of Pathology, Forensic Medicine, Institute of Biomedical Sciences, Faculty of Medicine, Vilnius University, Vilnius, Lithuania

**Keywords:** giant ovarian cyst, giant ovarian cystadenoma, Raktažodžiai: gigantinė kiaušidžių cista, gigantinė kiaušidžių cistadenoma

## Abstract

**Background:**

Cysts are called giant when they are >10 cm in diameter. Today the frequency of giant ovarian cysts is decreasing due to good diagnostic methods and regular gynecological examination. These cysts occur more frequently in women of reproductive and postmenopausal age. The most common giant cysts are benign serous cystadenomas. Diagnosing giant cysts can be hindered by nonspecific symptoms, the patient‘s reluctance to see a doctor and fear of surgery. The purpose of this study is to review the existing literature on this topic and describe three cases of giant cysts found at autopsy.

**Materials and methods:**

A literature review was conducted in the Medline (PubMed) database over a period of 10 years. The information concerning the examination of deceased individuals after their death was sourced from the database of the Lithuanian State Forensic Medicine Service. After the analysis of the deceased persons in the years 2015–2022 in the State Forensic Medicine Service, out of 1638 deceased persons, three cases of giant cysts were identified. These three cases were analyzed retrospectively. During these investigations, the circumstances of finding the scene, the results of the autopsy, and the data of additional toxicological and microscopic tests were evaluated.

**Results:**

A retrospective study of 3 cases confirms the fact that giant ovarian cysts are a rare phenomenon. In case 1, the corpse of a 75-year-old woman was examined, a 30x30x25 cm left ovarian cyst was found, the woman had chronic ischemic heart disease; case 2 was a 65-year-old woman with a 19x25x12 cm right ovarian cyst and deep vein thrombosis with pulmonary embolism; case 3 was a 62-year-old woman with a 40x30x30 cm right ovarian cyst and chronic ischemic heart disease. In all cases, giant ovarian cysts were incidental findings and not the primary cause of death. The described clinical cases corresponded to the characteristics of giant cysts indicated in the literature: appeared in the postmenopausal period, benign course, diagnosed in patients who did not seek medical attention.

**Conclusions:**

Thanks to good early diagnosis, ovarian cysts are diagnosed early, before they reach gigantic size, so giant cysts are extremely rare. Delayed diagnosis is related to reluctance of patients to consult doctors and confusion with ascites and obesity. They are usually benign, and the symptoms they cause are related to mass effect – pressure on nearby organs.

## Abstract (it)

**Įvadas:**

Cistos vadinamos gigantinėmis, kai jų skersmuo >10 cm. Šiandien gigantinių kiaušidžių cistų dažnumas mažėja dėl gerų diagnostikos metodų ir reguliarios ginekologinės patikros. Šios cistos dažniau atsiranda reprodukcinio ir pomenopauzinio amžiaus moterims. Dažniausios gigantinės cistos yra gerybinės serozinės cistadenomos. Diagnozuoti gigantines cistas gali trukdyti nespecifiniai simptomai, pacientės nenoras kreiptis į gydytoją ir operacijos baimė. Šio tyrimo tiks-las – apžvelgti esamą literatūrą šia tema ir aprašyti tris gigantinių cistų, aptiktų autopsijos metu, atvejus.

**Metodai:**

Atlikta *Medline (PubMed)* duomenų bazės 10 metų literatūros apžvalga. Informacija apie mirusių asmenų apžiūrą po jų mirties paimta iš Lietuvos valstybinės teismo medicinos tarnybos duomenų bazės. Valstybinėje teismo medicinos tarnyboje 2015–2022 metais atlikus mirusių asmenų analizę, iš 1638 mirusių asmenų nustatyti trys gigantinių cistų atvejai. Šie trys atvejai analizuoti retrospektyviai. Tyrimų metu buvo vertinamos įvykio vietos radimo aplinkybės, autopsijos rezultatai, papildomų toksikologinių bei mikroskopinių tyrimų duomenys.

**Rezultatai:**

Atliktas retrospektyvus 3 atvejų tyrimas patvirtina faktą, kad gigantinės kiaušidžių cistos yra retas reiškinys. 1 atvejo metu tirtas 75 metų moters lavonas, rasta 30×30×25 cm kairiosios kiaušidės cista, moteris sirgo lėtine išemine širdies liga; 2 atvejis – 65 metų moteris, turinti 19×25×12 cm dešinės kiaušidės cistą ir giliųjų venų trombozę su plaučių embolija; 3 atvejis – 62 metų moteris su 40×30×30 cm dešinės kiaušidės cista ir lėtine išemine širdies liga. Visais atvejais gigantinės kiaušidžių cistos buvo atsitiktiniai radiniai, ne pagrindinė mirties priežastis. Aprašyti klinikiniai atvejai atitiko literatūroje nurodytas gigantinių cistų charakteristikas: atsirado pomenopauziniu laikotarpiu, gerybinės eigos, diagnozuotos pacientėms, kurios nesikreipė į medikus.

**Išvada:**

Dėl geros ankstyvos diagnostikos kiaušidžių cistos diagnozuojamos anksti, dar nespėjus pasiekti gigantinio dydžio, todėl gigantinės cistos yra itin retos. Pavėluota diagnozė yra susijusi su pacienčių nenoru kreiptis į gydytojus ir klaidingai diagnozuotu ascitu ar nutukimu. Dažniausiai kiaušidžių cistos būna gerybinės, o sukeliami simptomai yra susiję su masės efektu – spaudimu į šalia esančius organus.

## Introduction

Cysts are called giant when they are >10 cm in diameter [1]. In the early days ovarian cysts used to reach remarkable sizes, one of the biggest ever recorded cysts weighed 148.6 kg [2]. Nowadays, there is a huge availability of various imaging techniques and patients usually do regular physical check-ups, for that reason giant ovarian cyst cases have become more uncommon [3]. The most common giant cysts are benign serous cystadenomas [4, 5]. It can be seen that ovarian cysts may occur at any age, but are more common at reproductive age [6]. Ovarian mucinous tumors are generally unilateral and benign, originating from epithelial tissue [7]. The diagnosis of giant cysts can be difficult due to nonspecific and late-appearing symptoms. Additionally, the reluctance of patients to consult doctors and the fear of surgery are also very significant. The purpose of this study is to review the existing literature on this topic and describe three autopsy cases of giant cysts that show that even with today’s good early diagnostic methods, they still occur.

## Materials and methods

### Study design and data source

**Fig. 1:**
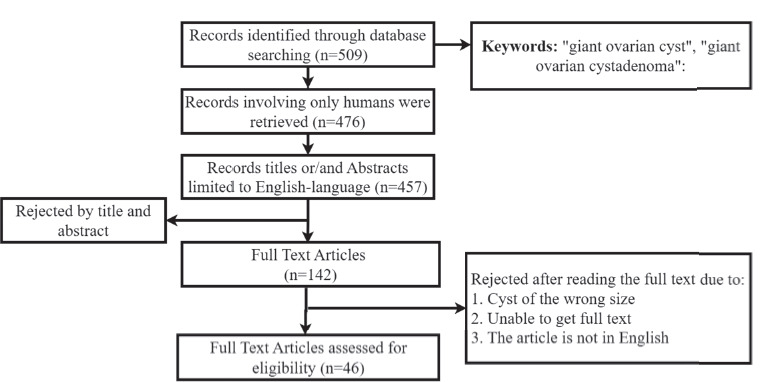
Literature review flowchart

A literature review was conducted in the Medline (PubMed) database over 10 years (Fig. 1). A detailed search, including the keywords “giant ovarian cyst,” “giant ovarian cystadenoma” has revealed a total of 509 records. A total of 476 articles involving only humans were retrieved. The search, which was limited to English-language publications, included a total of 457 articles published between 2010 and 2024, of which 46 articles were relevant to this research.

### Identification of cases

The performed retrospective study of 3 cases confirms the fact that giant ovarian cysts are a rare phenomenon. The described clinical cases correspond to the characteristics found in the literature: appeared in the postmenopausal period, benign course of the giant cyst, diagnosed in patients who did not seek medical attention.

The information concerning the examination of deceased individuals after their death was sourced from the database of the Lithuanian State Forensic Medicine Service. After the analysis of the deceased persons in the years 2015–2022 in the State Forensic Medicine Service, out of 1638 deceased persons, three cases were identified in which the ovarian cysts measured greater than 10 cm in diameter and met the criteria for a giant ovarian cyst. A full-body autopsy was performed on all deceased individuals to thoroughly examine the circumstances surrounding their death. During the autopsy, blood and urine samples were systematically collected for alcohol, medications and drug testing.

### Toxicological methods

After the forensic dissection, blood and urine samples were collected for alcohol and drug tests. Headspace gas chromatography was used to detect the presence of alcohol, while liquid chromatography-time-of-flight mass spectrometry (LC/MS-TOF) and chromatography-tandem mass spectrometry (LC-MS/MS) were used for quantitative drug detection.

### Histological methods

First, histological sections were prepared for routine light microscopy. Histomorphological features of the samples were examined by hematoxylin and eosin (H&E) staining. Next, Perl’s Prussian blue reaction was used to detect ferric iron and Masson’s trichrome staining of the collagen fibers. The nucleus and other DNA/RNA-containing structures were dyed blue-violet color while the cytoplasm and matrix were dyed pink.

## Case reports

### Case 1

**Fig. 2:**
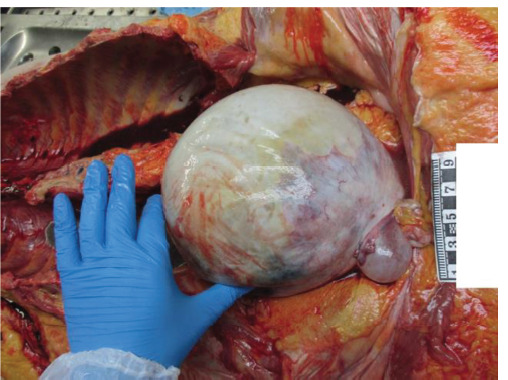
Macroscopic view of the ovarian cyst. Case 1

A corpse of a 75-year-old woman was found in the courtyard, sitting by the staircase door with her back against the wall and the metal cellar door. After examining the body, no external signs of violence or mechanical injuries were observed. An internal examination of the corpse showed that there were no bruises on the soft tissue. The peritoneum was grayish in color, smooth, and shiny, with 1500 ml of yellowish liquid in its cavity. The primary cause of death was chronic ischemic heart disease: atherosclerosis of the coronary vessels, stenosis over 75%, cardiac and large vessel valve fibrosis, uneven heart muscle perfusion, focal fibrosis, and a postinfarction scar in the anterior left ventricle wall. Histology showed interstitial fibrosis with cardiomyocyte hypertrophy. Concomitant diseases were atherosclerosis of the aorta, renal sclerosis, renal cysts, ovarian tumor with necrosis, soft tissue fibrosis of the brain, and atherosclerosis of the arteries, stenosis up to 25%. An internal examination revealed a 30 × 30 × 25 cm ovarian cyst with a wall thickness of 0.3 cm, and 2250 ml of grayish cloudy secretion was found in its lumen (Fig. 2). Additionally, blood and urine toxicological tests were performed; however, the results were negative.

### Case 2

**Fig. 3:**
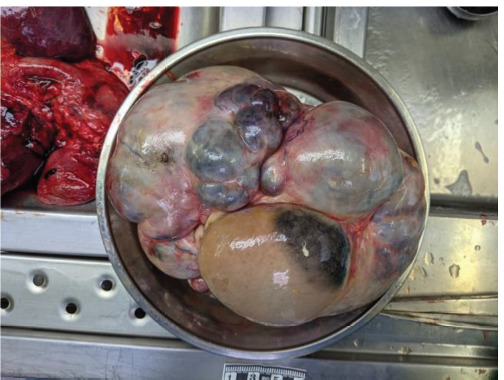
Macroscopic view of the ovarian cyst. Case 2

A corpse of a 65-year-old female was found at home. There were no external mechanical injuries. After conducting an internal examination of the corpse, no bruises on the soft tissue were found. The peritoneum exhibited a matte appearance in certain areas and had a greenish hue, with approximately 150 ml of brownish fluid present in the cavity. The intestine was swollen, and the intestinal loops were loose. The main cause of death was deep vein thrombosis of both calves and hamstrings: popliteal and femoral. Complications included pulmonary embolism. Additional concurrent diseases noted were: coronary artery atherosclerosis with stenosis reaching 95% on both sides; alongside aortic atherosclerosis; fatty liver; soft tissue fibrosis in the brain and pancreas; right atrial thrombus formation of the right lung and left kidney cysts. An internal examination revealed a right ovary measuring 19 × 25 × 12 cm and weighing 3308 g (Fig. 3). It consisted of multichambered structures that were filled with yellowish transparent liquids, brownish-green liquids, white masses, and yellowish pus-like liquid. The left ovary was 3 × 5 × 3 cm in size with a cyst of 3 cm in diameter, filled with a brownish cloudy liquid. Additionally, blood and urine toxicological tests were performed; however, the results were negative.

### Case 3

**Fig. 4:**
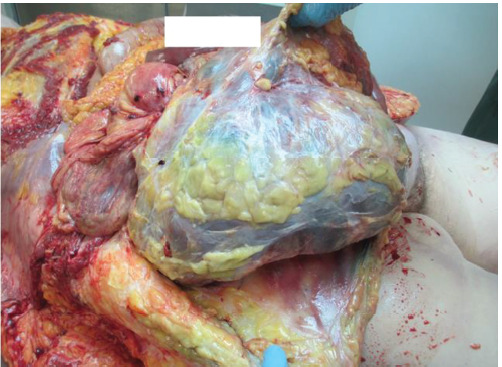
Macroscopic view of the ovarian cyst. Case 3

A corpse of a 62-year-old female was found in the house, lying naked on her back on the bed and covered with a blanket. The deceased did not visit doctors, nor did she receive treatment and only took blood pressure medication. There were no external mechanical injuries to the body. An internal examination of the corpse was carried out: no bruises on the soft tissue were found. The main cause of death was chronic ischemic heart disease: coronary artery atherosclerosis, stenosis up to 75%, and cardiac muscle fibrosis. Concomitant diseases: aortic atherosclerosis, fatty liver, soft tissue fibrosis of the brain. A right ovarian cyst measuring 40 × 30 × 30 cm was found, containing a brownish cloudy secretion in its cavity (Fig. 4). The left ovary was unchanged. Additionally, blood and urine toxicological tests were performed; however, the results were negative.

The findings of these three cases are summarized in Table 1. In all three cases, the cause of cadaveric death was unrelated to giant ovarian cysts.

**Table 1:** Three autopsy cases in which giant cysts were found.

	*Case 1*	*Case 2*	*Case 3*
*Gender*	Female	Female	Female
*Age*	75	65	62
*Location of corpse*	Near the staircase	Home	Home
*Ovarian cyst type*	Glandular structures of cylindrical light atypical cells.	Multichambered structures that are filled with liquids.	Multichambered structures that are filled with brownish cloudy liquids.
*Ovarian cyst location*	Left ovary	Right ovary	Right ovary
*Ovarian cyst size*	30 × 30 × 25 cm	19 × 25 × 12 cm	40 × 30 × 30 cm
*Histological examination*	Ovarian cystadenomas		

## Discussion

Giant ovarian cysts are very rarely occurring masses [4]. The literature contains numerous documented cases and studies on this topic. Cysts are called giant when they are >10 cm in diameter or if the size of the cyst surpasses the umbilicus [1]. Due to routine gynecological examination and visual diagnostic methods, cysts are usually detected at a small size, but if not noticed in time, they can become gigantic [3]. Unfortunately, there are still some cases of giant ovarian cystadenomas, that weigh over 100 kg [8]. The earliest diagnosed giant cyst was in a third-trimester fetus, which was a serous cystadenoma [9]. Ovarian serous cystadenomas, benign tumors originating from the ovarian epithelium, are the most common type of ovarian cysts, accounting for 75% of cases, while mucinous cyst adenomas account for the remaining 25% [4, 5]. Ovarian mucinous tumors are generally unilateral and benign, originating from epithelial tissue. They are lined with tall columnar, nonciliated epithelial cells that have apical mucin and basal nuclei [7]. They are usually benign (80%), their diameter ranges from 15 to 30 cm and the majority of women do not feel any symptoms in early stages and they account for 15% of all ovarian tumors [8, 10]. The age of patients varies from babies [11, 12] and adolescents [13, 14] to adults [15] including postmenopausal women [16], it can also occur during pregnancy [17]. Giant ovarian cysts account for less than 1% of cysts related to pregnancy, and their symptoms are nonspecific [18]. It can be seen that ovarian cysts may occur at any age, but are more common at reproductive age [6]. Mucinous cystadenomas are most frequently found in individuals between their “thirties” and “sixties” and are rarely diagnosed in very young or old people [3]. These cysts cause no symptoms at early stages, with clinical symptoms occurring only after the cyst becomes massive [19]. The most common clinical symptoms of ovarian cysts typically include increasing abdominal tension, nonspecific diffuse abdominal pain, vaginal bleeding, and symptoms related to organ compression such as constipation, early satiety, vomiting, and frequent urination [2]. There also might be some unusual symptoms such as left-sided back pain followed by acute left leg swelling [20]. A sudden onset of pain primarily located in the lower back and right flank, radiating to the hypogastric region [21]. Leg swelling and cough might also be the symptoms [20, 22].

Imaging is crucial in diagnosis. For evaluating large cysts transabdominal ultrasound (US) is better than the endo-vaginal US [23] and while US is useful, computed tomography (CT) and magnetic resonance imaging (MRI) (which is better than CT) are the best methods for analyzing the cyst. CT is more sensitive yet less specific than US in detecting ovarian cysts [18, 23]. Aspiration of giant ovarian cysts should not be used for diagnostics [2].

### Cancer markers

Women with a high-risk family history of ovarian cancer should undergo early screening with transvaginal ultrasound and serum CA 125, which could help detect this benign adenoma earlier [3]. CA 125 is mainly a marker for epithelial ovarian carcinoma, but it is elevated in only 50% of early-stage cases. While CA 125 is frequently studied as a serum marker for evaluating the likelihood of malignant tumors in adnexal masses, it is not reliable for distinguishing between benign and malignant ovarian masses, especially in premenopausal women [10]. There are cases where serum concentration of CA 125 was higher but pathologic findings of ovarian cysts were benign. It is known that the serum level of CA 125 may be higher in gynecologic malignancies as well as in other conditions such as benign ovarian neoplasms, functional ovarian cysts, pelvic inflammatory diseases, pregnancy, and menstruation [24]. Cancer markers such as β-human chorionic gonadotropin, CA 19-9, CA 125, and CAE are usually not elevated in benign cysts [21, 23, 25, 26], but in few cases, CA 125 [27–29] and CA 19-9 [16] was found. HE4 (marker for ovarian cancer diagnosis) is not expressed in normal ovarian cells but is highly expressed in ovarian cancer [23].

### Delayed Diagnosis and Management of Giant Ovarian Cysts

The diagnosis of cysts is usually delayed. The most common causes of delayed diagnosis are associated with obesity [30–33] and misdiagnosed ascites [2, 26, 29, 34–37]. Other causes are a cyst masked by pregnancy [18, 38], mixed with a cyst of abdominal organs [39]. Less common manifestations of a giant cyst are intestinal compartment syndrome due to pressure caused by a giant cyst [16] and hydronephrosis [24].

Giant ovarian cysts are commonly mistaken for ascites in postmenopausal women due to their large size. This misdiagnosis occurs because the physical examination findings and symptoms of giant ovarian cysts can resemble symptoms of ascites [2]. Ascites have also been mistakenly diagnosed in pregnant patients with giant ovarian cysts [40]. The timeframe for seeking medical help for this tumor varies due to different symptoms, willingness to seek care, and economic status. Diagnoses can be missed due to obesity or self-neglect. Fear of a cancer diagnosis also contributes [3]. The literature describes a case where parents do not seek medical attention because they think their child has gained weight, so they have no willingness to seek medical care [34]. In another case described in the literature, the diagnosis was delayed due to the patient’s low socio-economic and educational status, which led the patient to believe that she was gaining weight [19]. During the COVID-19 pandemic in 2020, access to primary care services was restricted and numerous general practitioners were consulting patients over the phone. This led to the inability to perform physical examinations and make good differential diagnoses, which made the diagnosis of giant ovarian cysts delayed [20]. A differential diagnosis should be considered for any abdominal cyst to avoid misdiagnosis, especially if the cyst is very large [39].

Clinicians need to differentiate giant ovarian cysts from other conditions. Possible reasons for an abdominal mass encompass benign and malignant factors from gynecological and nongynecological sources [18]. Ovarian cysts can range from simple and functional cysts to malignant neoplasms. The differential diagnosis list is extensive and includes paraovarian cysts, appendiceal mucocele, cystic adenomyosis, liver or pancreatic cysts, lymphocele, bladder diverticulum, pelvic endometriosis, intraabdominal pregnancy, and more [19, 23]. Before considering surgical procedures, it is important to rule out hypothyroidism in girls with multicystic ovaries [41].

Complications can include cyst torsion, hemorrhage, rupture of the adnexal mass, and sometimes even death. Acute life-threatening complications may involve pleural effusion, small bowel obstruction, and venous thromboembolism [3].

Patients with adnexal torsion exhibit varying clinical manifestations due to differences in tumor characteristics and the extent of blood flow obstruction, making misdiagnosis and missed diagnosis common. Accurate and timely diagnosis, along with surgical treatment, is crucial to prevent irreversible damage and preserve ovarian function, particularly for women who wish to have children in the future [10, 15].

Surgical management is the best treatment for extra-large ovarian cysts [42]. Due to the compressive symptoms and potential for malignancy, surgery is essential for patients with giant ovarian cysts, with laparotomic removal and intraoperative pathological assessment being the standard treatment method [19].

### Autopsy findings of giant ovarian cysts

The current autopsy case studies support the idea that many women overlook the signs of ovarian tumors, primarily due to societal issues and a lack of awareness regarding giant cysts. Raising awareness and understanding of giant ovarian tumors is crucial, along with highlighting the significance of early detection [43]. Our cases described above illustrate that patients with symptoms of a giant ovarian cyst, such as an increase in the size of the abdomen, do not seek medical attention. The incidental discovery of giant ovarian cysts during autopsies is a rare phenomenon. In reviewing the literature, similar findings have been reported in various case studies, where cysts often went undiagnosed for a long period due to the lack of symptoms or because they were confused with other conditions. A case highlighted by Chute et al. details a middle-aged woman who passed away at home as a result of the enlargement of a mucinous cystadenoma in her left ovary. Three years before her death, she exhibited symptoms of an undiagnosed giant cyst, which was incorrectly identified as ascites. After extracting nearly 26 liters of fluid, the cyst’s dimensions were measured at 35.5 × 23 × 20 cm. The large ovarian cyst increased abdominal pressure and pushed her diaphragm upward. After the autopsy, the cause of death was certified as giant left ovary cystadenoma complications [26]. Kashiwagi et al. reported the death of a woman due to an ovarian tumor with ascites. The autopsy revealed a right ovarian tumor measuring 40.0 × 41.5 × 19.0 cm. The total weight of the right ovary and uterus was 13.0 kg. The tumor and ascites caused circulatory failure due to increased intra-abdominal pressure [44]. Duran et al. reported a patient who died of terminal ileum obstruction and necrosis caused by compression of a giant ovarian mass measuring 11 × 10 × 7 cm and weighing 382 g [45]. Suzuki et al. reported a rare autopsy case of an ovarian tumor diagnosed as a giant 5 kg (24 cm in diameter) mucinous cystic tumor complicated by bacterial abscess infection [46].

In contrast to the literature, where giant ovarian cysts were directly linked to fatal outcomes, the cases in our study involved incidental findings of cysts during autopsy, with the cause of death unrelated to the cysts. Additionally, unlike the literature cases where misdiagnosis played a critical role, the cysts in our cases were not misdiagnosed before death.

## Conclusions

Thanks to good early diagnosis, ovarian cysts are diagnosed early before they reach gigantic size, so giant cysts are extremely rare. Delayed diagnosis is related to the reluctance of patients to consult doctors and confusion with ascites and obesity. Most ovarian cysts are benign and the symptoms they cause are related to mass effect – pressure on nearby organs.
